# Antibody-Dependent Enhancement of SARS-CoV-2 Infection of Human Immune Cells: In Vitro Assessment Provides Insight in COVID-19 Pathogenesis

**DOI:** 10.3390/v13122483

**Published:** 2021-12-11

**Authors:** Xu-Rui Shen, Qian Li, Hui-Ling Li, Xi Wang, Qi Wang, Xiao-Shuang Zheng, Rong Geng, Yu-Lan Zhang, Bei Li, Ren-Di Jiang, Mei-Qin Liu, Yan Zhu, Wei Zhang, Xing-Lou Yang, Ke Peng, Peng Zhou

**Affiliations:** 1 CAS Key Laboratory of Special Pathogens and State Key Laboratory of Virology, Wuhan Institute of Virology, 430071 Wuhan, China; pukulovesu@163.com (X.-R.S.); therealliqian@163.com (Q.L.); huilingli18@163.com (H.-L.L.); wx_nwafu@163.com (X.W.); wangqi@wh.iov.cn (Q.W.); hixszheng@163.com (X.-S.Z.); gengrong_11@163.com (R.G.); zhangyl@wh.iov.cn (Y.-L.Z.); libei@wh.iov.cn (B.L.); drteamwind1004@163.com (R.-D.J.); liumq126@163.com (M.-Q.L.); zhuyan@wh.iov.cn (Y.Z.); zhangwei@wh.iov.cn (W.Z.); yangxl@wh.iov.cn (X.-L.Y.); 2 University of Chinese Academy of Sciences, 100049 Beijing, China

**Keywords:** SARS-CoV-2, antibody-dependent enhancement, COVID-19 pathogenesis, convalescent serum, excessive immune response

## Abstract

Patients with COVID-19 generally raise antibodies against SARS-CoV-2 following infection, and the antibody level is positively correlated to the severity of disease. Whether the viral antibodies exacerbate COVID-19 through antibody-dependent enhancement (ADE) is still not fully understood. Here, we conducted in vitro assessment of whether convalescent serum enhanced SARS-CoV-2 infection or induced excessive immune responses in immune cells. Our data revealed that SARS-CoV-2 infection of primary B cells, macrophages and monocytes, which express variable levels of FcγR, could be enhanced by convalescent serum from COVID-19 patients. We also determined the factors associated with ADE, and found which showed a time-dependent but not viral-dose dependent manner. Furthermore, the ADE effect is not associated with the neutralizing titer or RBD antibody level when testing serum samples collected from different patients. However, it is higher in a medium level than low or high dilutions in a given sample that showed ADE effect, which is similar to dengue. Finally, we demonstrated more viral genes or dysregulated host immune gene expression under ADE conditions compared to the no-serum infection group. Collectively, our study provides insight into the understanding of an association of high viral antibody titer and severe lung pathology in severe patients with COVID-19.

## 1. Introduction

COVID-19, which is caused by SARS-CoV-2, poses a great threat to public health and the global economy [1]. Patients with COVID-19 generally raise antibodies against SARS-CoV-2 following infection, and the antibody level is positively correlated to the severity of disease [2]. Although it was believed that antibodies, particularly neutralizing antibodies played a pivotal role in inhibiting SARS-CoV-2 replication in patients, it has also been argued that they may also exacerbate COVID-19 through antibody-dependent enhancement (ADE) [3]. 

ADE has been documented to other viruses including dengue virus (DENV), respiratory syncytial virus (RSV), measles virus, and feline infectious peritonitis virus (FIPV) [4,5,6,7]. In these cases, ADE increased the severity of diseases either by enhanced antibody-mediated virus uptake into Fc gamma receptor (FcγR)-expressing phagocytic cells, leading to increased viral infection and replication (type I ADE), or by excessive antibody Fc-mediated effector functions or immune complex formation causing enhanced inflammation and immunopathology (type II ADE) [3]. The type I ADE normally requires viral productive infection of target immune cells, for example, macrophages or monocytes in the case of FIPV in cats [6]. The type II ADE can occur without the need of viral productive infection albeit causing dysregulated immune activation of target cells [3]. In humans, FcγR is expressed broadly among the various leukocyte subsets including macrophages, monocytes, B cells, and others, and modulates downstream immune responses upon binding to the Fc domain of an IgG antibody [8]. Subsequently, these leukocytes could be potential targets of virus induced ADE. 

ADE has been well characterized in cats infected with FIPV, a feline betacoronavirus. Experimental infection of FIPV antibody positive cats resulted in more severe diseases, regardless of naturally acquired or vaccine acquired antibodies [6]. This ADE is also closely related to more viral replication and more inflammatory responses in viral target cells including monocytes and macrophages in an aminopeptidase N (APN)-independent, FcγR-dependent manner [9,10]. Likewise, it is not unexpected that SARS-CoV-1 and MERS-CoV could induce ADE in FcγR-expression Raji B cells or HEK293T cells in vitro [11,12]. It is also postulated that SARS-CoV-2 may also induce ADE in some leukocytes.

Severe patients with COVID-19 normally generated high levels of SARS-CoV-2 antibodies, and the antibody titer was positively related to the severity of disease, which showed less neutralization potency [13]. This phenomenon suggests that ADE induced by non-neutralizing antibodies could play an important role in the pathogenesis of SARS-CoV-2 in patients. Moreover, it was argued that immune cells, which normally express low or no ACE2 receptors, could also be infected. Viral RNA positive or antigen positive have been reported in a few single-cell analysis of patient BALF or in postmortem analysis of COVID-19 patients. Whether this positivity is caused by ADE or a direct ACE2-independent infection is still unknown.

In this study, we tested SARS-CoV-2 induced ADE in vitro using convalescent COVID-19 patient serum samples in a list of FcγR-expression leukocytes. Our results contributed to the understanding of the pathogenesis of SARS-CoV-2 in the context of viral treatment and control.

## 2. Materials and Methods

### 2.1. Primary Immune Cells Preparation and SARS-CoV-2 Infection

This study obtained informed consent from all subjects. The blood samples from healthy donors were treated with Ficoll-Paque Plus (17144002; Cytiva, Danaher Corporation, WDC, USA). Briefly, 3 mL of Ficoll was added to a centrifuge tube, then 4 mL of whole blood was gently added before being centrifuged at 400× *g* for 30 min at 20 °C. The PBMC layer was carefully taken with a pipette. Magnetic beads conjugated with different cell markers were used to sort immune cells: CD19 microbeads (130-050-301; Miltenyi Biotec--, Bergisch Gladbach, Germany) for B cells, CD14 microbeads for monocytes (130-050-201; Miltenyi Biotec, --Bergisch Gladbach, Germany), and CD11b-beads for macrophages (130-049-601; Miltenyi Biotec, --Bergisch Gladbach, Germany). Primary immune cells after sorting were cultured in Roswell Park Memorial Institute 1640 culture medium (RPMI1640, C22400500BT; Thermo Fisher Scientific, Waltham, MA, USA) supplemented with 10% fetal bovine serum (FBS, 10099141; Life Technologies, Thermo Fisher Scientific, Waltham, MA, USA ).

For infection, primary B cells, monocytes, and macrophages were seeded into 24-well plates or 48-well plates at a density of 1 × 10^6^ cells/mL. Cells were infected by SARS-CoV-2 at a moi of 0.1. 0 h samples were harvested once mixed cells with virus. Cells were washed three times using RPMI1640 and harvested for qRT-PCR or flow cytometry detection. Remaining cells were cultured at 37 °C supplied with 5% CO_2_ for 24 h or 48 h before collecting for further analysis.

For detection of antibody-dependent enhancement (ADE), virus (moi = 0.1) was incubated with equal-volume convalescent sera from COVID-19 patients (for no-sera group, virus were incubated with equal-volume RPMI1640) at 37 °C for 30 min. Mixture were added to primary cells and samples were harvested at 0 h, 24 h, or 48 h post infection.

### 2.2. Cell Lines and Virus Culture

Primary B cell, monocyte, macrophage, and Raji in RPMI-1640 (C22400500BT; Thermo Fisher Scientific, Waltham, MA, USA) + 10% FBS (10099141; Life Technologies, Thermo Fisher Scientific, Waltham, MA, USA), or Vero E6 and Caco-2 in DMEM + 10% FBS (Gibco, C 11995500BT) were cultured at 37 °C in a humidified atmosphere of 5% CO_2_. All cell lines were tested free of mycoplasma contamination and applied to species identification and authenticated by microscopic morphologic evaluation. None of the cell lines was on the list of commonly misidentified cell lines (by ICLAC). The SARS-CoV-2 isolate WIV04 (GISAID accession number EPI_ISL_402124) was used in this study. WIV04 was isolated from Huh7 cells from the original sample and was passaged in Caco-2 cells. Viral titer (TCID50/mL) was determined in Vero E6 cells.

### 2.3. Proteins and Antibodies for SARS-CoV-2

NP and predicted RBD of SARS-CoV-2 strain WIV04 were inserted into pCAGGS vector with an N-terminal S-tag. Constructed plasmids were transiently transfected into HEK293T-17 cells. The supernatant collected for protein purification was purified using S-tag resin and the purity and yield was tested using anti-S-tag mAb (generated in-house). Rabbits were immunized with purified NP proteins or RBD protein three times at a dose of 700 ng/each at a two week interval. Rabbit serum was collected at 10 days after the final injection. Antibody titer was determined in an ELISA using purified NP protein or RBD protein as the detection antigen.

### 2.4. B Cell Line Infection

Raji B cells were infected with SARS-CoV-2 at a moi of 0.01, 0.1, or 0.2 depending on the purpose of the experiment. Infected cells were harvested at 0, 24, or 48 h after three washes with RPMI1640. Cellular viral RNA or sg-RNA expression was determined in qPCR or RNA-Seq. GAPDH was used in qPCR as the internal control. For detection of antibody-dependent enhancement (ADE), virus (moi = 0.01, 0.1, or 0.2) were incubated with equal-volume convalescent sera from COVID-19 patients (for no-sera group, virus were incubated with equal-volume RPMI1640) at 37 °C for 30 min. Mixture was added to primary cells and samples were harvested at 0 h, 24 h, or 48 h post infection.

### 2.5. Flow Cytometry Analysis of Human Peripheral Blood Samples

For FCGRs detection, primary B cells, monocytes, and macrophages were incubated with fluorochrome-labeled antibodies specific for humans before fixation: FITC mouse anti-human CD32 (552883; BD Pharmingen, San Diego, CA, USA), APC mouse anti-human CD64 (561189; BD Pharmingen, San Diego, CA, USA ), CD16 Rabbit PAb (16559-1-AP; Proteintech,--). FITC-anti-Rabbit IgG (H+L) (SA00003-2; Proteintech, Chicago, IL, USA) was used as the secondary antibody for CD16.

For SARS-CoV-2 infected primary immune cells, surface staining was conducted before fixation with AF700-anti-CD45 (368514; BioLegend, San Diege, CA, USA), BV650-anti-CD11b (101239; BioLegend, San Diege, CA, USA), PE-anti-CD68 (333808; Biolegend, San Diege, CA, USA), and Percp Cy5.5-anti-CD14 (367110; Biolegend, San Diege, CA, USA). Antibody stained cells were fixed overnight with 4% PFA at 4 °C and taken out of the BSL3 lab for downstream analysis. Cells were stained further with in house made SARS-CoV NP pAb (1:500) at 4 °C for 30 min after permeabilization. Then, cells were stained with FITC-anti-Rabbit IgG (H + L) (SA00003-2; Proteintech, Chicago, IL, USA) at room temperature for 30 min.

### 2.6. RNA Extraction and qRT-PCR

Whenever commercial kits were used, the manufacturer’s instructions were followed without modification. Viral RNA was extracted from 140 μL of samples with the QIAamp^®^ Vival RNA Mini Kit (52906; QIAGEN, Hilden, Germany). RNA was eluted in 50 μL of elution buffer and used as the template for qRT–PCR. The QPCR detection method based on the 2019-nCoV S gene can be found in the previous study (Zhou et al., 2020). Two microliters of RNA were used as a template for the amplification of selected genes by real-time quantitative PCR using HiSxript^®^ II One step qRT-PCR SYBR^®^ Green Kit. (Q221-01; Vazyme Biotech Co., Ltd, Nanjing, Jiangsu, China). The 10 μL qPCR reaction mix contained 1.9 μL of nuclease free water, 5 μL of 2× One Step SYBR Green Mix, 0.5 μL One Step SYBR Green Enzyme Mix, 0.2 μL of 50× ROX Reference Dye 1, 0.2 μL of each primer (10 μM), and 2 μL of template RNA. Amplification was performed as follows: 50 °C for 3 min, 95 °C for 30 s followed by 40 cycles consisting of 95 °C for 10 s, 60 °C for 30 s, and a default melting curve step in an Step-One Plus Real-time PCR machine (ABI) [14].

### 2.7. Transcriptome Analysis

Using software Hisat2 v2.1.0, raw reads was mapped to genome that combined with human GRCH38.913 and Sars-CoV2 (MN996528.1). After transforming the format and sorting in Samtools v1.10-24, the BAM file was passed to StringTie v2.1.0 for transcriptome assembly and quantitation. Read count table of transcriptome generated by prepDE.py, a tool in StringTie, was used for gene differential expression analysis in R v4.1.0 with package DESeq2 v1.32.0. Compared to the mock group, the gene with Log2 Fold Change >2 and *p*-value < 0.05 was retained. Furthermore, the genes whose expression increased with degree of ADE were passed to the online tool Metascape for enrichment analysis.

### 2.8. Micro-Neutralization Assay

For detection of the neutralization antibody titer of convalescent sera, SARS-CoV-2 were diluted to 4000 TCID50/mL and incubated with equal-volume diluted patient sera (diluted from 1:10 to 1:1280, two-fold serial dilution) at 37 °C for 30 min. This was added to the mixture of Vero E6 cells seeded in 96-well plates and infected at 37 °C for 1 h. The supernatant was removed and cells washed with PBS. DMEM medium containing 2% FBS were added to the cells. Cell plates were fixed at 24 h post infection. Stained cells with in house made SARS-CoV NP pAb (1:500) and Cy3-anti-Rabbit IgG (H+L) were from Proteintech (SA00009-2) to detect viral NP.

### 2.9. Serological Test

An in-house anti-SARS-CoV-2 IgG ELISA kit was developed using recombinant RBD of the SARS-CoV-2 isolate WIV04 (MN996528.1). The RBD proteins were expressed in HEK293-17 cell lines. For IgG analysis, MaxiSorp Nunc-immuno 96-well ELISA plates were coated (100 ng per well) overnight at room temperature with RBD protein. Plasma from different donors were used at a dilution of 1:20 for 1 h at 37 °C. A HRP-conjugated anti-human IgG monoclonal antibody (Kyab Biotech Co. Ltd., Wuhan, China) was used at a dilution of 1:40,000. The OD value (450–630 nm) was calculated.

### 2.10. Statistical Analysis

Data analyses were performed using GraphPad Prism 7.0 software. Data are shown as mean ± SD. Data were analyzed with the Shapiro–Wilk normality test and confirmed with the Gaussian distribution. Statistical analysis was performed using the Student’s *t*-test with two tailed, 95% confidence. *p* values less than 0.05 were considered statistically significant.

## 3. Results

### 3.1. Convalescent Sera from COVID-19 Patients Induced ADE in Primary Peripheral Blood Immune Cells In Vitro

**Figure 1:**
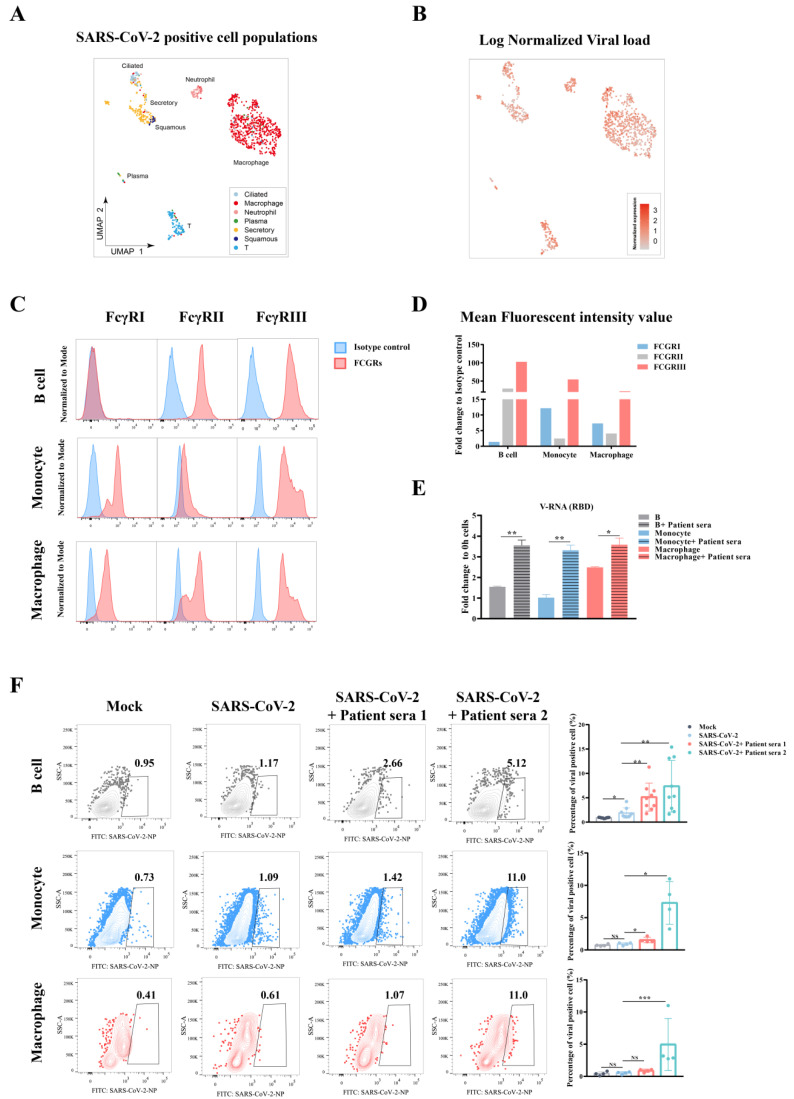
Convalescent sera from COVID-19 patients induced ADE in multiple peripheral blood immune cells. (**A**) UMAP show the overview of the population of SARS-CoV-2 RNA positive cells (*n* = 1078), which were derived from BALF and two sputum samples of COVID-19 patients. Identification of cell population was corresponded to the description of a previous study [15]. (**B**) Normalized expression of SARS-CoV-2 RNA in each virus positive cell. Data were reanalyzed from the published dataset [15]. (**C**) Expression of different FcγRs including FcγRI, FcγRII, and FcγRIII on primary B cells, monocytes, or macrophages was detected by flow cytometry. (**D**) Quantification of FcγR expression by comparing the mean fluorescent intensity value. (**E**) SARS-CoV-2 virus pretreated with equal-volume convalescent sera from one COVID-19 patient or RPMI1640 medium at 37 °C for 30 min. Viral–serum mixtures were added to primary B cells, monocytes, and macrophages that were prepared from one healthy donor at a moi of 0.1. Samples were harvested at 48 h post infection and cellular viral load was then quantified by qRT-PCR detection targeting the viral receptor-binding domain (RBD). (**F**) SARS-CoV-2 virus was pretreated with equal-volume convalescent sera from two COVID-19 patients (RBD IgG OD values of 1.759 and 1.851, respectively) or RPMI1640 medium at 37 °C for 30 min. Viral–serum mixtures were added to primary B cells, monocytes, and macrophages from healthy donors at a moi of 0.1. Samples were harvested at 48 h post infection and cellular viral NP was then detected by flow cytometry. The data were analyzed using the Student’s t test and statistical significance is indicated (* *p* < 0.05; ** *p* < 0.01; *** *p* < 0.001; NS, no significance).

It has been demonstrated that multiple immune cell types collected from COVID-19 patients harbor SARS-CoV-2 viral RNA, suggesting they could be actively engulfed virus or they could have been infected [15]. Here, we reanalyzed the data from a previous single-cell RNA-Seq study and found the presence of viral RNA of SARS-CoV-2 in multiple cell types from bronchoalveolar lavage fluid (BALF) and sputum samples [15]. These infected cells include respiratory tissue cells such as ciliated cells, secretory cells, and squamous cells as well as immune cells including macrophages, neutrophils, plasma B cells, and T lymphocytes (Figure 1A,B).

As leukocytes co-express low or no ACE2 or TMPRSS2 [15], the main entry mechanism of SARS-CoV-2, we then determined if they could be targets of viral induced ADE, which was based on the surface expression of FcγR and the presence of viral antibodies [16]. Peripheral blood cells were collected from healthy donors and detected for the expression of CD64 (FcγRI), CD32 (FcγRII), and CD16 (FcγRIII) in primary B cells, monocytes, and macrophages using flow cytometry. The three cell types were chosen as they not only showed viral positive in patients, but are also important antigen presenting cells that play an important role in adaptive immunity. Our results showed that these primary immune cells express two or three kind of FcγRs, suggesting they could be targets of ADE (Figure 1C).

To detect whether ADE could occur on these FcγR-expression immune cells, primary B cells, monocytes, and macrophages were enriched by microbeads (Supplementary Figure S1A) and then infected with SARS-CoV-2 at a moi of 0.1. Before infection, we incubated the virus with culture medium or convalescent sera from a COVID-19 patient. We detected viral RNA in three primary immune cells by qRT-PCR. Our results showed an around two to three-fold increase in viral RNA in B cells or monocytes, and 1-fold increase in macrophages in sera treated cells compared to the control, indicating more viral infection in the presence of convalescent sera from COVID-19 patients (Figure 1D,E)

To further corroborate these findings, we tested this sera-enhanced viral infection using flow cytometry. We pre-incubated SARS-CoV-2 with sera from two COVID-19 patients that have different antibody titers and then detected viral NP by flow cytometry. Our results showed a minimum viral positivity in these immune cells without serum, whereas the addition of COVID-19 patient serum greatly enhanced viral infection by as much as a 7-fold increase in B cells and monocytes, or 5-fold increase in macrophages. Moreover, the second patient serum with a higher titer of viral antibody than the first serum demonstrated better efficacy in the context of enhancing viral infection (Figure 1F). Taken together, SARS-CoV-2 infection of immune cells was enhanced in the presence of convalescent sera from COVID-19 patients, suggesting ADE.

### 3.2. Factors That Determined ADE Effect In Vitro

**Figure 2:**
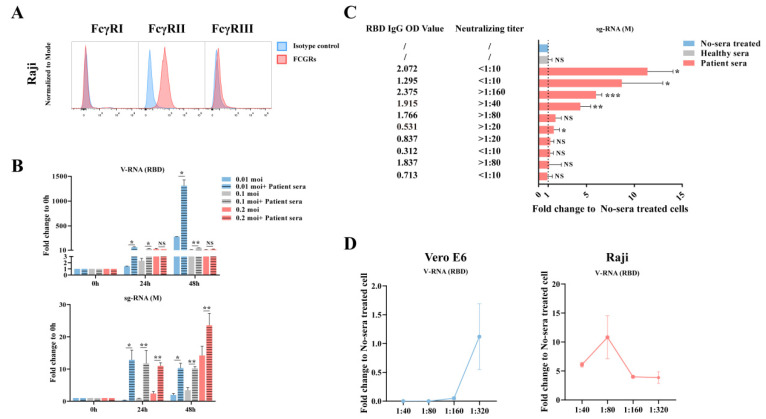
Factors related to ADE effect. (**A**) The expression of FcγRs in Raji B cells, a cloned immortalized cell line. (**B**) Raji B cells were infected with SARS-CoV-2 at a moi of 0.01, 0.1, and 0.2 with or without convalescent sera. Samples were harvested at 0 h, 24 h, or 48 h post infection and cellular viral load was then quantified by qRT-PCR detection of total viral RNA or subgenomic RNA (sgRNA). (**C**) SARS-CoV-2 virus pretreated with equal-volume convalescent sera from different COVID-19 patients (1:40 of final dilution) or RPMI1640 at 37 °C for 30 min. Viral mixtures were added to Raji B cells at a moi of 0.1. Samples were harvested at 48 h post infection and cellular viral load was then quantified by qRT-PCR detection of sgRNA. RBD IgG OD value and neutralizing titer of convalescent sera were quantified by ELISA or micro-neutralization assay, respectively. (**D**) Convalescent serum from one COVID-19 patient was diluted to 1:40, 1:80, 1:160, and 1:320. SARS-CoV-2 was pre-incubated with equal-volume sera at 37 °C for 30 min and then added to Vero E6 or Raji B cells at a moi of 0.1. Samples were harvested at 48 h post infection and cellular viral load was then quantified by qRT-PCR detection of viral RBD. The Vero E6 detection data can be found in Supplementary Figure S1B. Comparison between different sample groups was analyzed by the Student’s *t*-test. * *p*<0.05; ** *p* < 0.01; *** *p* < 0.001; NS, no significance. The short lines mean to compare viral load in infected cells with or without patient sera).

Since we observed the ADE effect during SARS-CoV-2 infection of primary immune cells, we then explored the factors that determined the level of ADE. For this purpose, we used a cloned B cell, Raji cell, for the downstream analysis considering the high ADE effect in primary B cells. Raji cells, which express the FcγRII receptor and a little FcγRIII receptor (Figure 2A), have been shown as a good model for testing SARS-CoV-1 induced ADE in vitro [11]. The Raji cells were infected with SARS-CoV-2 at a moi = 0.01, 0.1, and 0.2 and harvested at 0, 24, 48 hpi with or without COVID-19 patient serum. The qRT-PCR results showed that the serum greatly enhanced viral infection in a time-dependent manner, similar to what has been observed in primary B cells (Figure 2B). We did not find a viral dose-dependent ADE effect here. However, this could be due to that viral infection was already saturated at a moi = 0.01 in Raji cells. Coronavirus generated a large amount of sub-genomic RNA (sgRNA) during cell replication, which was then used as an indicator of replicating virus [17]. We then determined the levels of sgRNA following infection. Similarly, more sgRNA was observed in the serum treated group, suggesting more replicating viruses in the ADE group (Figure 2B).

COVID-19 patients normally generated different levels of SARS-CoV-2 antibodies, and more severe patients appear to have higher antibody titers [13]. Whether a higher antibody contributed to disease severity due to the ADE effect was debated. We then tested this theory in vitro using convalescent sera collected from 10 COVID-19 patients. These serum samples have variable levels of RBD IgG titer or SARS-CoV-2 neutralizing titers, as determined in RBD ELISA or in Vero E6 cells following infection (Supplementary Figure S1B). We diluted these sera to 1:40 and incubated with SARS-CoV-2 before infection of Raji cells. The qRT-PCR results showed that at least four of the convalescent sera induced ADE, although the effect was not associated with RBD antibody titer or neutralizing titer (Figure 2C). Notably, two of the serum samples that had higher than 1:40 neutralizing titer showed full protection in Vero E6 cells but induced ADE in Raji cells, whereas the other two ADE inducing samples had high RBD antibody titer, but low neutralizing titer.

In a study investigating ADE of severe dengue disease in humans, the risk of severe dengue disease was found to be highest within a narrow range of preexisting anti-DENV antibody titers [4]. We then determined whether the antibody titer also determined SARS-CoV-2 ADE effect in Raji cells. We pre-incubated SARS-CoV-2 with multiple dilutions of an ADE-induced convalescent serum from 1:40 to 1:320 before infection. Cellular viral sgRNA was detected by qRT-PCR. Our results indicated that the serum sample completely inhibited viral replication in Vero E6 cells when the dilution was 1:40 and 1:80, but slowly lost this neutralizing activity at 1:160 and 1:320. In contrast, none of the dilutions neutralized viral infection of Raji, but caused significant ADE in all dilutions. Notably, ADE effect was most obvious in a medium level of 1:80-dilution and decreased following dilution, which was similar to ADE in dengue viral disease (Figure 2D).

### 3.3. Antibody-Dependent Enhancement of Excessive Activation of the Immune Cascade

None-neutralizing antibodies produced by patients may enhance disease and immunopathology by excessive Fc-mediated effector functions and immune complex formation in an antibody-dependent manner. Previous studies suggest that the most probable ADE mechanism relevant to COVID-19 pathology is the formation of antibody–antigen immune complexes, which leads to excessive activation of the immune cascade in lung tissue [3].

**Figure 3:**
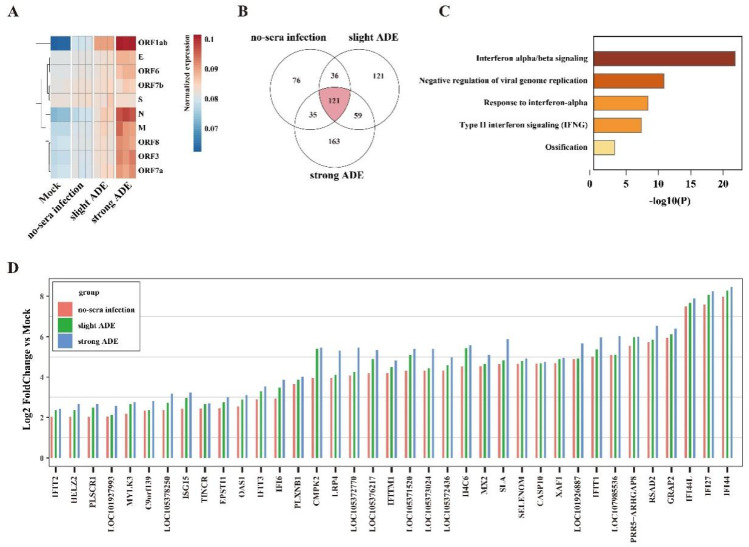
Antibody-dependent enhancement of excessive immune activation. Mock or SARS-CoV-2 infected Raji B cells with or without convalescent sera were harvested at 48 h post infection and subjected for RNA-seq analysis. (**A**) Heatmap showing the correlation of different groups and the viral genes that were enriched in samples compared to mock cells. (**B**) Common upregulated genes and respective upregulated genes of no-sera infection, slight ADE, and strong ADE group. (**C**) GO showing the most significant upregulated genes in ADE group cells compared to the no-sera treated infected cells. (**D**) Log2 fold change of the commonly upregulated genes in no-sera infection, slight ADE, or strong ADE groups compared to the mock infected group.

We next determined whether convalescent sera from COVID-19 patients could also enhance host immune activation. We tested the cellular viral or host gene expression in Raji B cells upon SARS-CoV-2 infection by bulk RNA-Seq analysis. In comparison, two serum samples that were known to induce slight or strong ADE in Figure 2 were used. Consistent with the qRT-PCR results, the expression level of all viral genes in the serum-treated group was higher in ADE groups, and stronger ADE was also followed by better higher expression (Figure 3A).

Finally, we ought to determine the immune responses following ADE. In a differential gene expression analysis, we determined 45 genes that were mutually upregulated in all three infected groups cells compared to mock infection (Figure 3B). In a Gene Ontology analysis, we found that the antiviral interferon related GO pathways were significantly upregulated including the “interferon alpha/beta signaling pathway”, “negative regulation of viral genome replication”, “response to interferon-alpha pathway”, and “type II interferon signaling pathway” (Figure 3C). When compared to the no-serum infection group, nearly all gene expression was upregulated in the serum-infection group, particularly in the strong-ADE group. Collectively, our data indicated that convalescent serum samples from COVID-19 patients caused excessive activation of the immune cascade (Figure 3D).

## 4. Discussion

Here, we tested the ADE effect of convalescent serum samples using in vitro immune cells, aiming for a better understanding of possible SARS-CoV-2 viral antibody induced pathology in vivo. Our data indicated that ADE could occur to FcγR-expression cells such as B cells, monocytes, or macrophages. Although the ADE effect was not correlated to the dose of antibody in a particular patient, it was found to be highest within a narrow range of preexisting titer in the serum that was known to induce ADE. Finally, the ADE effect includes not only an enhancement of viral replication, but also an excessive immune response in these immune cells.

The role of SARS-CoV-2 antibodies in the severity of diseases is controversial. Based on the observations that severe patients tend to have higher antibody titers, there have been two hypotheses: one possibility is that the extensive viral replication and hyper-inflammation in severe patients induced overproduction of antibodies, while another possibility is that the high levels of antibodies worsen disease severity via ADE. The hyper-inflammation, however, was a result of ADE [3]. In this study, we showed the in vitro evidence of ADE in multiple peripheral blood immune cells, supporting the second hypothesis that non-neutralizing antibodies could worsen the disease severity by enhancing viral infection or enhancing excessive immune activation in certain immune cells, suggesting they contributed to SARS-CoV-2 induced immunopathology in severe patients.

The ADE effect has been well characterized in cat coronavirus FIPV infection. FIPV infected macrophages and monocytes via APN receptor, and this infection or infection induced host responses were greatly enhanced by pre-existing native antibodies in cats [6,9,10]. Likewise, a list of in vitro experiments also proved that SARS-CoV-1 or MERS-CoV induced ADE, although there is a lack of in vivo evidence [11,12]. Therefore, it has been suggested that strong antibody titers are more closely linked to severe COVID-19, which could be important in the lower respiratory tract that contributes to lung pathology [3]. Our data proved the theory that more viral infection and more excessive immune responses in B cells, macrophages, and monocytes, the three cell types that are heavily recruited in lung under severe condition, and thus would increase the severity in lung. Moreover, a medium level of antibody can more easily induce ADE, a phenomenon that is also observed in dengue disease [4], suggesting that the patients with antibody titers at or near the peak enhancement titer may place these individuals at greater risk of severe disease than if they only developed a small amount of antibody. Finally, the ADE effect may further dampen our immune defense mechanism by causing the dysfunction of B cells or macrophages, which eventually leads to impaired adaptive immunity.

Our study also has some limitations. First, this in vitro study provides insight into the pathogenesis of COVID-19 enhanced by antibodies. However, we were unable to provide in vivo data in the pathogenesis of a more severe clinical outcome in patients or in experimental animals because the effector functions of antibodies are altered by species–species interactions between antibodies and immune cells [16]. Second, our study may not apply to predict potential ADE effect upon vaccination. It was revealed that SARS-CoV-2 natural infection induced a list of “bad antibodies” that would not show up after vaccination, for example, autoantibodies or lower levels of fucosylation of SARS-CoV-2-specific antibodies [18]. The IgG types may also vary between infection and vaccination.

Collectively, we showed in vitro evidence of convalescent serum-dependent enhancement of either SARS-CoV-2 infection or viral induced excessive immune responses in immune cells. Our study provides insights into the understanding of an association of high viral antibody titer and severe lung pathology in severe patients with COVID-19.

## Data Availability

The data presented in this study are available on request from the corresponding author. The data are not publicly available due to limitations in material transfer agreement.

## Supplementary Materials

The following are available online at https://www.mdpi.com/article/10.3390/v13122483/s1, Figure S1: Preparation of primary immune cells and the neutralizing activity of convalescent serum samples in Vero E6 cells.
