# Supplementary material for: Antibody-Dependent Enhancement of SARS-CoV-2 Infection of Human Immune Cells: In Vitro Assessment Provides Insight in COVID-19 Pathogenesis
Source: Viruses. 2021 Dec 11;13(12):2483. doi: 10.3390/v13122483 (PMC8704563; doi:10.3390/v13122483)
Supplement: Supplementary file 1 [file viruses-13-02483-s001.zip › viruses-1485966-supplementary.pdf]

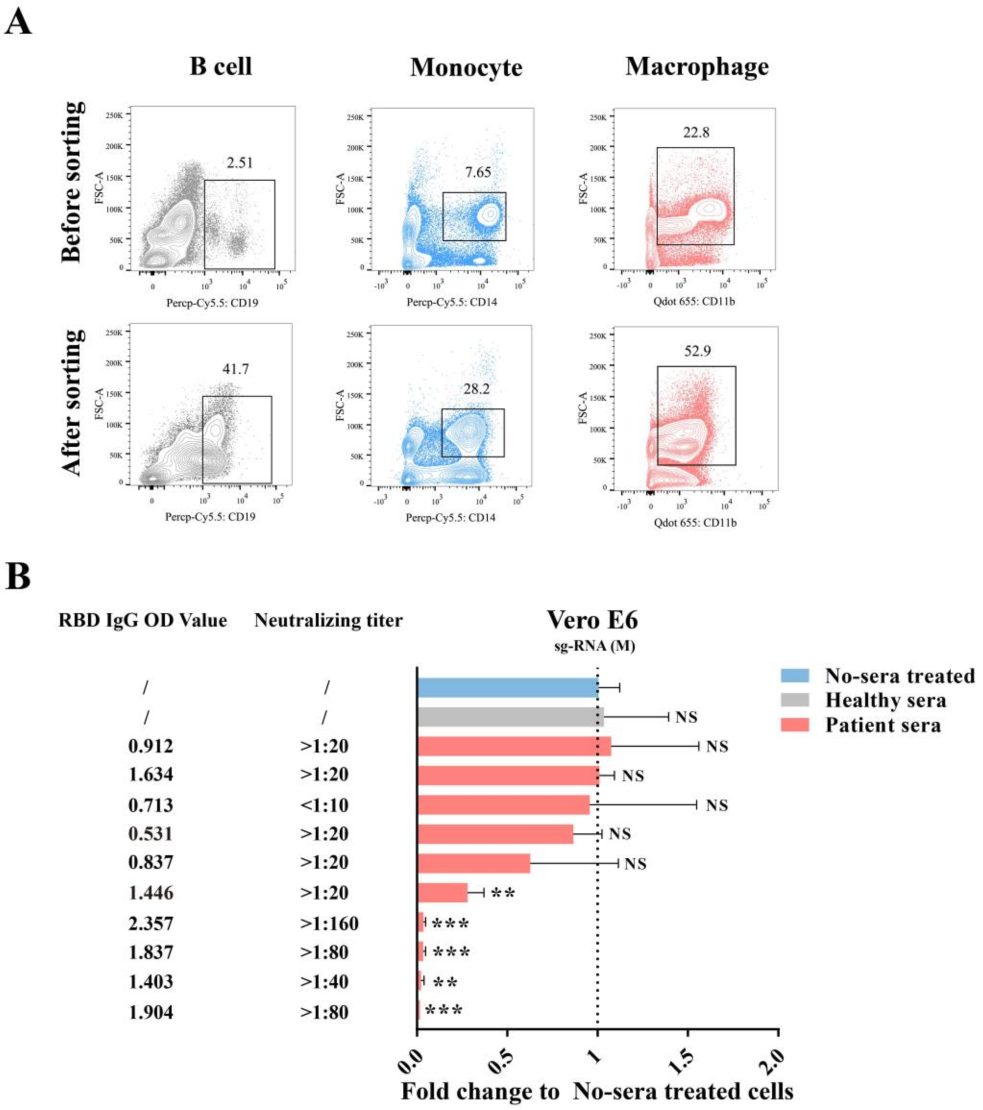

Supplementary Figure S1. Preparation of primary immune cells and the neutralizing activity of convalescent serum samples in Vero E6 cells. (**A**) Enrichment of peripheral immune cells from healthy donors. Primary B cells, monocytes and macrophages were enriched by microbeads from PBMCs. The target cells in PBMCs before and after sorting were compared. (**B**) Neutralizing titers in Vero E6 cells. SARS-CoV-2 virus pretreated with equal-volume of convalescent sera from 10 COVID-19 patients (1:40 of final dilution), sera from a healthy donor or RPMI1640 at 37℃ for 30 min. Viral mixtures were added to Vero E6 cells at an moi of 0.1. Samples were harvested at 48h post infection and cellular viral load was quantified by qRT-PCR detection of sub-genomic RNA. RBD IgG OD value and neutralizing titer of convalescent sera were quantified by ELISA and micro neutralization assay respectively.
